# The reference range of lamotrigine in the treatment of epilepsy in children: a systematic review

**DOI:** 10.1007/s00228-023-03562-9

**Published:** 2023-10-31

**Authors:** Jingjing Chen, Liang Huang, Linan Zeng, Zhimei Jiang, Meiping Xiong, Zhi-Jun Jia, Guo Cheng, Liyan Miao, Limei Zhao, Lingli Zhang

**Affiliations:** 1 Department of Pharmacy, West China Second University Hospital, Sichuan University, Chengdu, China; 2 Evidence-Based Pharmacy Center, West China Second University Hospital, Sichuan University, Chengdu, China; 3 NMPA Key Laboratory for Technical Research on Drug Products In Vitro and In Vivo Correlation, Chengdu, China; 4 Key Laboratory of Birth Defects and Related Diseases of Women and Children, Sichuan University, Ministry of Education, Chengdu, China; 5 West China School of Pharmacy, Sichuan University, Chengdu, China; 6 Department of Pediatrics, West China Second University Hospital, Sichuan University, Chengdu, China; 7 Laboratory of Molecular Translational Medicine, Center for Translational Medicine, Sichuan University, Chengdu, China; 8 Department of Pharmacy, The First Affiliated Hospital of Soochow University, Suzhou, China; 9 Department of Pharmacy, Shengjing Hospital of China Medical University, Shenyang, China; 10 Chinese Evidence-Based Medicine Center, West China Hospital, Sichuan University, Chengdu, China

**Keywords:** Lamotrigine, Concentration, Reference range, Therapeutic drug monitoring, Child, Epilepsy

## Abstract

**Purpose:**

This study intends to assess the reference range of lamotrigine concentration for treating childhood epilepsy.

**Methods:**

PubMed, Ovid-Embase, The Cochrane Library, CNKI, WanFang data and VIP databases were searched from database inception to January 2022. RCT, cohort study, case–control study, cross-sectional study that estimated the reference range of lamotrigine for children epilepsy treatment were included. The data extracted included basic information, statistical methods, data type, and results of reference range. Descriptive analysis was performed for them.

**Results:**

8 studies were included and estimated the reference range, and all of them were calculated based on efficacy data and/or concentration data. Statistical methods including ROC curve, concentration-effect curve, mean ± standard deviation, 95% confidence interval and percentile interval were utilized. For lamotrigine monotherapy, the lower limits ranged from 2.06 mg/L to 3.99 mg/L, and the upper limits ranged from 8.43 mg/L to 9.08 mg/L, showing basic consistency. However, for lamotrigine concomitant with valproate, the lower limits ranged from 2.00 mg/L to 8.00 mg/L, and the upper limit was 11.50 mg/L, for lamotrigine concomitant with other antiepileptics, the lower limits ranged from 1.00 mg/L to 3.09 mg/L, and the upper limits varied from 5.90 mg/L to 16.24 mg/L, indicating inconsistency.

**Conclusion:**

Several studies have estimated the reference range of lamotrigine for childhood epilepsy, while controversy exist and no studies have determined the upper limit of the range based on safety data. To establish the optimal reference range, further high-quality studies are necessary that consider both efficacy and safety data.

**Supplementary Information:**

The online version contains supplementary material available at 10.1007/s00228-023-03562-9.

## Introduction

Epilepsy is a prevalent neurological disease among children, with an incidence rate of approximately 33/100000–82/100000 per year [1]. The primary approach to managing seizures, reducing hospitalization, incidence, and mortality rate, and improving learning ability and quality of life is through antiseizure medications (ASMs) therapy [2]. Lamotrigine (LTG) is a new ASM with high oral bioavailability, a long half-life time, and a minimal impact on liver and kidney function. It is considered a first-line ASM in China for newly diagnosed child tonic–clonic seizures, tonic seizures and absence seizure [3]. However, pharmacokinetics of LTG may be influenced by several factors such as age, gender, concomitant medication, liver/kidney function, and gene polymorphism, and inter-individual variability may also exist [4]. It is recommended to maintain an optimal concentration level to minimize adverse effects and optimize treatment outcomes, because excessive drug concentration is relatively more likely to cause adverse effects, while a concentration that is too low may result in treatment failure [5]. Children are a special population, and required concentrations are always different from adults, while therapeutic drug monitoring (TDM) guidelines of International League Against Epilepsy (ILAE) and Arbeitsgemeinschaft für Neuropsychopharmakologie und Pharmakopsychiatrie (AGNP) have recommended the reference concentration (2.5 or 3 mg/L to 15 mg/L) for the use of LTG in adult epilepsy treatment, there is no recommendation for children [4, 6]. This study aims to provide more credible evidence for the individualization of LTG in childhood epilepsy treatment by systematically evaluating clinical studies on the reference concentration of LTG in children with epilepsy.

## Materials and methods

This study complied with the PRISMA Statement [7].

### Inclusion criteria

#### Participants

Children aged 0–18 years and diagnosed with epilepsy [8].

#### Interventions and controls

LTG monotherapy or LTG concomitant with valproate (VPA) or other ASMs.

#### Outcomes

Reference range and synonym or similar terms such as concentration reference range, therapeutic reference range, therapeutic range, optimal range, effective range, target range and orienting range.

#### Types of studies

Randomized controlled trial (RCT), cohort study, case–control study, cross-sectional study that estimated the reference range of LTG for children.

### Exclusion criteria

Duplicate publications, data not available, full-text not available.

### Definition

Reference range: In our study, we used the term “reference range” to refer to the range of concentrations that were referred to as concentration range, therapeutic reference range, therapeutic range, optimal range, effective range, target range, and orienting range in the included studies.

Therapeutic regimen: In this study, LTG therapeutic regimen were classified as 3 types: LTG monotherapy, LTG concomitant with VPA, and LTG concomitant with other ASMs. Other ASMs refers to ASMs that are not LTG or VPA. LTG concomitant with VPA is when patients are only treated with LTG and VPA without any other ASMs. LTG concomitant with other ASMs is when patients are treated with LTG and other ASMs, with or without VPA.

Data type: the purpose of this term is to determine which types of data were utilized in determining the reference concentration. We classified data into three types: concentration data, efficacy data and safety data. Concentration data refers to the concentration level of LTG. Efficacy data evaluates treatment effects, such as clinical response rates and seizure reduction rate (RR). Safety data evaluates the safety of LTG, including adverse effects rate, incidence of toxicity and incidence of liver injury.

### Retrieval strategy

PubMed, Ovid-Embase, The Cochrane Library, CNKI, WanFang data and VIP databases were searched from inception to January 2022. For additional studies, the reference of reviews was also checked. The retrieval strategy is shown in Online Resource 1.

### Study selection and data extraction

2 researchers independently screened and selected studies. Firstly, titles and abstracts were screened to determine the potential studies that meet the criteria. Then, full texts were assessed. A third researcher resolve any existed disagreements. Data was also independently extracted by 2 researchers using Excel form. Information including basic information of the studies (titles, years, countries, authors, types of study), basic information of participants (age, gender, epilepsy type), sample size, LTG therapeutic regime and data of reference range (method of estimation, types of data and result of reference range, including upper and lower limit).

### Quality assessment

To date, there are no standardized quality tools for studies specifically investigating therapeutic drug monitoring (TDM), we evaluated the quality of the studies based on the recommendation of Hart et al. [9]. For RCTs, we used the Cochrane Handbook recommended ROB2 tool assessing the risk of bias [10].For cohort study and case–control study, we used the Newcastle–Ottawa Quality Assessment Scale (NOS) assessing the risk of bias [9, 11]. For cross-sectional study, we used the checklist recommended by Agency for Healthcare Research and Quality (AHRQ) assessing the quality, for item 5, If “NO” or “Unclear” was answered, this item would be scored “1”; If “Yes” was answered, this item would be scored “0”. The rules are opposite for other items. Studies with a score of 0–3 were assessed as low quality, 4–7 were assessed as moderate quality, and 8–11 as high quality [12]. In addition, we also used a quality tool specifically designed for TDM studies to assess their quality [9]. The quality score ranges from 0 to 10, with higher scores indicating higher quality.

### Statistical analysis

We performed descriptive analysis in this study.

## Results

### Search and study selection

**Fig.1:**
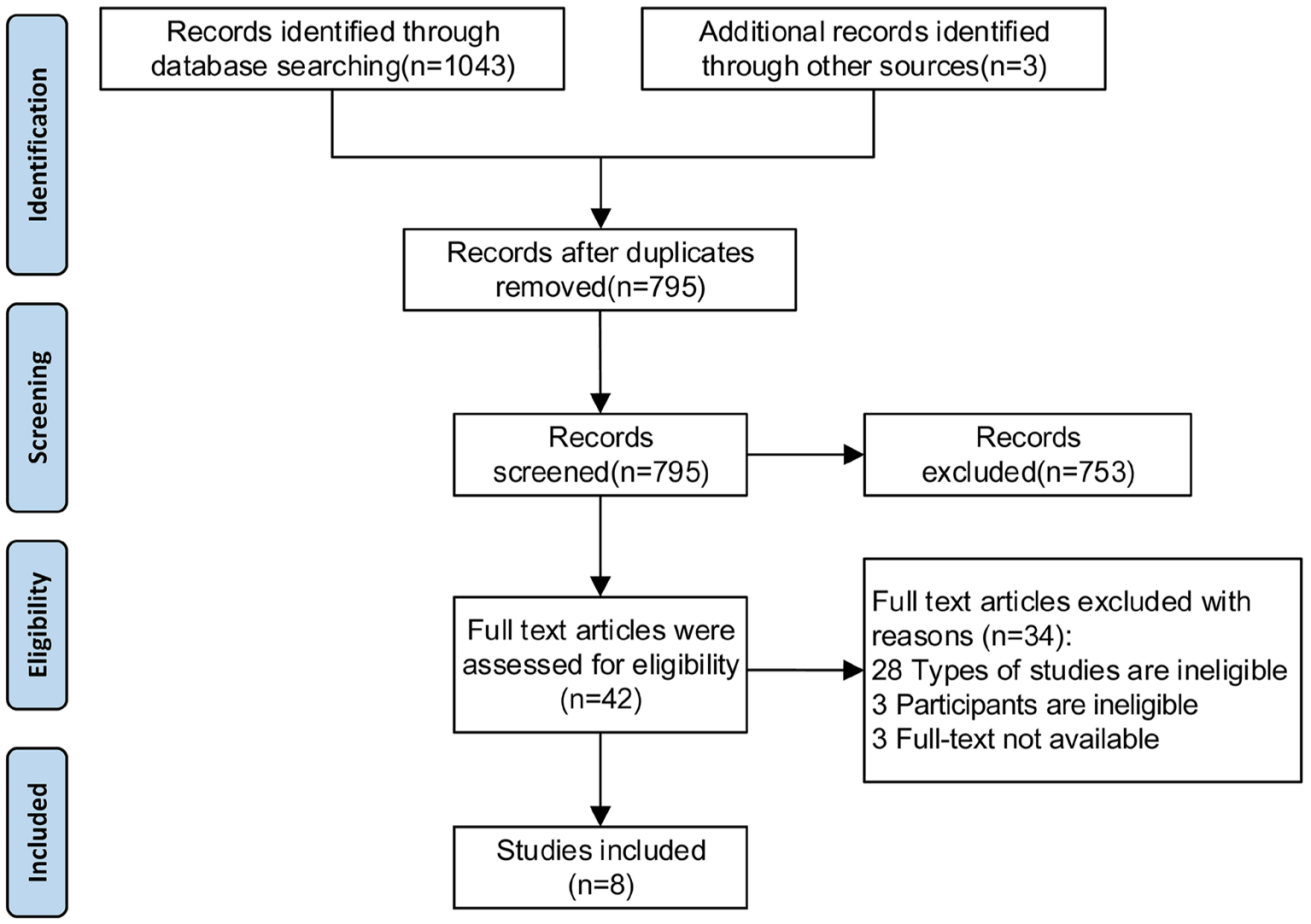
Study flow diagram

A total of 1043 studies were identified initially, only 8 cross-sectional study met the criteria and were included in our study [13–20] , no RCT, cohort study or case-control study was included (Fig. 1).

### Study quality assessment

Two tools were used to assess the quality of all studies: the AHRQ checklist for cross-sectional study and a quality tool for TDM studies. According to the AHRQ checklist, 7 studies were determined to be of moderate quality, while 1 study was deemed to be of high quality. According to the quality tool for TDM studies, 3 studies scored 8.35, 2 studies scored 7.35, and 3 studies scored 6.85 (Table 1).

**Table 1:** Characteristics of the included studies

**Author**	**Year**	**Country**	**Age (years, mean ± SD/median)**	**Sample size (n)**	**LTG therapeutic regimen**		**Concentration**			
					**Dosage**	**Combination therapy**	**Blood collection time**	**Concentration type**	**Quality 1**	**Quality 2**
Wang [16]	2019	China	7.73 ± 2.17^*^; 15.04 ± 2.19^**^	41^*^/69^**^	12.5–675 mg/d	Monotherapy	5–7 half-life periods after the start of LTG medication	Trough concentration	Moderate	8.35
Zhao et al. [17]	2019	China	9.91 ± 4.44	200	0.62–6.25 mg/kg/d	Monotherapy	≥ 3 months after reaching the maintenance dose of LTG	Trough concentration	Moderate	7.35
Wang et al. [20]	2021	China	8.48 ± 2.08^*^; 14.51 ± 2.02^**^	168^*^/171^**^	Not applicable	Monotherapy	≥ 4 weeks after reaching the maintenance dose of LTG, and every 3 months for the duration of the study	Trough concentration	High	8.35
Iwasaki et al. [19]	2015	Japan	9.16 ± 3.34	32	Initial dosage: 0.15 mg/kg/d, Maintenance dosage:1–3 mg/kg/d, limited to 200 mg/d	Concomitant with VPA	At 6 months, 1 year, and 2 years after reaching the maintenance dose of LTG	Peak concentration	Moderate	8.35
He et al. [15]	2017	China	8.70	72	1.33–2.48 mg/kg/d	Concomitant with VPA	After reaching the maintenance dose of LTG within 1 year	Trough concentration	Moderate	7.35
Bao et al. [13]	2001	China	8.40 ± 3.70	21	Initial dosage:0.5-1 mg/kg/d Maintenance dosage:2-15 mg /kg/d	Concomitant with other ASMs	8–20 days after reaching the maintenance dose of LTG	Trough concentration	Moderate	6.85
Li and Huang [14]	2014	China	10.15	111	4.01 ± 0.48 mg/kg/d	Concomitant with other ASMs	At 1 months after reaching the maintenance dose of LTG	Trough concentration	Moderate	6.85
Zhao et al. [18]	2021	China	9.05 ± 4.17	266	Initial dosage: 25 mg qd, Maintenance dosage:100–200 mg/d	Concomitant with other ASMs	≥ 3 months after reaching the maintenance dose of LTG	Trough concentration	Moderate	6.85

* Younger children (2- < 12 years); ** older children (12–18 years); Quality 1: evaluated by the AHRQ checklist for cross-sectional study; Quality 2: evaluated by the quality tool for TDM studies; LTG: lamotrigine; VPA: valproate; ASMs: antiseizure medications; AHRQ, Agency for Healthcare Research and Quality

### Study characteristics

8 Studies comprising 1151 children with epilepsy were included in this study. Studies were published from 2001 to 2021, two of them were retrospective study and 6 were retrospective study. Children in 7 studies were from China and 1 study were from Japan. 2 studies further grouped children into younger children (aged 2 to < 12 years) and older children (aged 12 to 18 years). Blood samples in all studies were collected after reaching the maintenance dose of LTG, 7 studies determined trough concentration, while 1 study determined peak concentration. Children in 3 studies received LTG monotherapy, 2 studies received LTG concomitant with VPA, and 3 studies received LTG concomitant with other ASMs. Details are shown in Table1.

### Estimation methods of reference range

2 studies only estimated the lower limit of reference range, and 6 studies estimated both upper limit and lower limit of reference range (Table 2).

**Table 2:** Calculating methods of reference range

**LTG therapeutic regimen**	**Research ID**	**Upper limit**					**Lower limit**					
		**ROC analysis**	**Concentration-effect curves**	**Mean ± standard deviation**	**95% confidence interval**	***Percentile interval***	**ROC analysis**	**Concentration-effect curves**	**Mean ± standard deviation**	**Chi-square test**	**95% confidence interval**	**Percentile interval**
LTG monotherapy	Zhao et al. [17]	/	/	/	/	/	√	/	/	/	/	/
	Wang [16]	/	√	/	/	/	√	/	/	/	/	/
	Wang et al. [20]	/	√	/	/	/	√	/	/	/	/	/
Concomitant with VPA	He et al. [15]	/	/	/	/	/	/	/	/	√	/	/
	Iwasaki et al. [19]	/	/	√	/	/	/	/	√	/	/	/
Concomitant with other ASMs	Li and Huang [14]	/	/	/	√	/	/	/	/	/	√	/
	Zhao et al. [18]	/	/	/	/	√	/	/	/	/	/	√
	Bao et al. [13]	/	/	/	√	/	/	/	/	/	√	/

*LTG* lamotrigine, *ROC* Receiver operating characteristic curve, *ASMs* antiseizure medications

#### LTG monotherapy

3 studies estimated reference range for LTG monotherapy. 2 of these studies estimated both the upper and lower limit concentrations, while1 study only estimated lower limit concentration.

For lower limit, 3 studies used the same method. Wang [16] and Wang et al. [20] enrolled 110 and 339 epileptic children aged 2–18 years respectively, and both divided children into two groups: (1) younger children (aged 2 to < 12 years) and (2) older children (aged 12 to 18 years). Zhao et al. [17] enrolled 200 epileptic children aged 0–18 years. In these 3 studies, children who experienced a reduction in seizure frequency (Seizure Reduction Rate, RR) of ≥ 50% compared to their baseline were classified as responders, while those who did not were classified as non-responders. The researchers established the lower limit concentration by performing a receiver operating characteristic curve (ROC), which based on the clinical response and LTG concentration.

For upper limit, both Wang [16] and Wang et al. [20] developed concentration-effect curves based on RR and concentration of LTG to calculate a threshold concentration beyond which therapeutic effect no longer increases significantly. This threshold concentration was confirmed as the upper limit. Zhao  [17] did not estimate upper limit.

#### LTG concomitant with VPA

2 studies estimated reference range for LTG concomitant with VPA [15, 19]. 1 of these studies estimated both the upper and lower limit concentrations [19], while 1 study only estimated lower limit concentration [15].

For lower limit, He et al. [15]enrolled 72 epileptic children treated with LTG concomitant with VPA. Children who experienced a RR of ≥ 50% compared to their baseline were classified as responders, while those who did not were classified as non-responders. In this study, children were divided into two groups based on a preset LTG level (≥ 2 μg/L or < 2 μg/L) and their clinical response was compared. The results showed that the non-responders in the < 2 μg/L group was obviously higher compared to the ≥ 2 μg/L group (χ^2^ = 5.3731, *P* = 0.02), and all patients in the ≥ 2 μg/L group were responders. Therefore, this study recommended the preset LTG level as a reference range for LTG in combination with VPA. Iwasaki et al. [19] enrolled 32 epileptic children aged 2.9–14.1 years that treated with LTG concomitant with VPA. Children who experienced a RR of 50% compared with baseline were defined as effective cases, and the others were defined as ineffective cases. This study discovered a significant difference in LTG blood concentrations between effective and ineffective cases. The concentration range in effective cases did not overlap with that of ineffective cases. The study confirmed the lower limit concentration as the average minus the standard deviation of concentrations in effective cases.

For upper limit, Iwasaki et al. [18] confirmed it as the average plus the standard deviation of concentrations in effective cases.

#### LTG concomitant with other ASMs

Bao et al. [13] included 44 epileptic children aged 3–14 years that treated with LTG concomitant with other ASMs. In this study, children who experienced a RR of ≥ 50% compared to their baseline were defined as effective cases, those who did not were classified as non-responders. The reference range was estimated by calculating the 95% confidence interval of concentration in all effective cases.

Li and Huang [14] included 111 epileptic children aged 3–18 years that treated with LTG concomitant with other ASMs. This study estimated the 95% confidence interval of concentration in all cases, and this 95% confidence interval was considered as the reference range.

Zhao et al. [18] included 266 epileptic children aged 0–18 years that treated with LTG concomitant with other ASMs. This study estimated the percentile interval (P2.5 to P97.5) of concentration in all cases, and this interval was considered as the reference range.

### Data type

6 studies estimated the reference range using efficacy data [13, 15–17, 19, 20], while 2 studies estimated the upper limit and lower limit of reference range only using concentration data [14, 18]. No studies used safety data.

#### LTG monotherapy

For LTG monotherapy, 3 studies estimated the upper and lower limit of reference range based on both concentration data and efficacy data. 2 of these studies [16, 20]assessed efficacy of LTG based on the average monthly number of epileptic seizures over 3 months, while time frame of another study was unclear [17] (Table 3).

**Table 3:** Methods of efficacy evaluation for studies that used efficacy data

**LTG therapeutic regimen**	**Research ID**	**Definition of responders**	**Definition of RR**	**Definition of A and B**
LTG monotherapy	Zhao et al. [17]	RR ≥ 50%	RR = (B-A)/B*100%	B: Monthly number of epileptic seizures at baseline A: Monthly number of at follow-up assessment
	Wang [16]	RR ≥ 50%	RR = (B-A)/B*100%	B: the average monthly number of epileptic seizures over the past 3 months before the start of LTG medication A: the average monthly seizure frequency over the past 3 months at the last follow-up assessment
	Wang et al. [20]	RR ≥ 50%	RR = (B-A)/B*100%	B: the average monthly number of epileptic seizures over the past 3 months before the start of LTG medication A: the average monthly seizure frequency over the past 3 months at follow-up assessment
Concomitant with VPA	He et al. [15]	RR ≥ 50%	RR = (B-A)/B*100%	B: Monthly number of epileptic seizures at baseline A: Monthly number of at follow-up assessment
	Iwasaki et al. [19]	RR > 50%	RR = (B-A)/B*100%	B: the seizure frequency for 28 days before the start of LTG medication; A: the seizure frequency for 28 days before the evaluation
Concomitant with other ASMs	Bao et al. [13]	RR > 50%	RR = (B-A)/B*100%	B: Monthly number of epileptic seizures at baseline A: Monthly number of at follow-up assessment

*RR* the rate of epileptic seizure frequency reduction, *LTG* lamotrigine, *ASMs* antiseizure medications

#### LTG concomitant with VPA

For LTG concomitant with VPA, 2 studies estimated the upper and lower limit of reference range based on both concentration data and efficacy data. One of these studies assessed efficacy of LTG based on the average monthly number of epileptic seizures over 28 days [19], however, time frame of another study was unclear [15] (Table 3).

#### LTG concomitant with other ASMs

For LTG concomitant with other ASMs, 2 studies estimated the upper and lower limit of reference range only using concentration data. 1 study used both concentration data and efficacy data, while time frame that assessed efficacy of LTG was unclear [13] (Table 3).

### Results of reference range

#### LTG monotherapy

Reference range recommended for LTG monotherapy in different studies were basically consistent. For younger children (aged 2 to < 12 years), Wang [16] recommended 3.99–8.97 mg/L and Wang ML recommended 3.29–9.08 mg/L. For older children (aged 12 to 18 years), Wang HX recommended 2.67–8.56 mg/L and Wang et al. [20] recommended 2.06–8.43 mg/L, which were lower than the recommendation for younger children. Zhao et al. [17] recommended ≥ 2.64 mg/L for children (Table 4).

**Table 4:** Results of reference range

**LTG therapeutic regimen**	**Research ID**	**Upper limit**	**Lower limit**	**Reference range (mg/L)**
LTG monotherapy	Zhao et al. [17]	/	√	≥ 2.64
	Wang [16]	√	√	Younger children^*^: 3.99–8.97; Older children^**^: 2.67–8.56
	Wang et al. [20]	√	√	Younger children^*^: 3.29–9.08; Older children^**^: 2.06–8.43
Concomitant with VPA	He et al. [15]	/	√	≥ 2.00
	Iwasaki et al. [19]	√	√	8.00–11.50
Concomitant with other ASMs	Li and Huang [14]	√	√	1.00–9.90
	Zhao et al. [18]	√	√	2.20–16.24
	Bao et al. [13]	√	√	3.09–5.90

^*^: 2- < 12 years; ^**^: 12–18 years; LTG: lamotrigine; VPA: valproate; ASMs: antiseizure medications

#### LTG concomitant with VPA

Reference range recommended for LTG concomitant with VPA varied across different studies. He et al. [15] recommended ≥ 2 mg/L, Iwasaki et al. [19] recommended 8–11.5 mg/L (Table 4).

#### LTG concomitant with other ASMs

Reference range recommended for LTG concomitant with other ASMs varied across different studies, they were 1–9.9 mg/L [19], 2.20–16.24 mg/L [18], and 3.09–5.90 mg/L [13] respectively (Table 4).

## Discussion

### Principle findings

To our knowledge, this study firstly provides a comprehensive summary of the reference range for LTG concentration in children, which offers credible evidence for the individualization of LTG treatment in epilepsy for this population. This study found that several studies have estimated reference concentration for LTG in child epilepsy treatment, but there still remains controversial. For LTG monotherapy, reference range were basically consistent. For younger children (2- < 12 years), the lower limit ranged from 3.29-3.99 mg/L, and the upper limit ranged from 8.97-9.08 mg/L. For older children (12-18 years), the lower limit ranged from 2.06-2.67 mg/L, and the upper limit ranged from 8.43-8.56 mg/L, indicating that and the lower limit of reference range for older children is similar to that recommended for adult by ILAE and AGNP guidelines (2.5 and 3 mg/L respectively), and younger children may require higher concentrations compared to older children and adults. However, the recommended reference range for LTG when concomitant with VPA or with other ASMs varied among different studies. The lower limit for LTG when concomitant with VPA recommended by Iwasaki et al. [19] was much higher than that of He et al. [15], mainly due to the difference in concentration type and calculating methods. He YL determined trough concentration, while Iwasaki determined peak concentration. The lower limit for LTG when concomitant with other ASMs ranged from 1.00-3.09 mg/L and the upper limit ranged from 5.90-16.24 mg/L, which may be due to the difference in combined ASMs, calculating methods and data type. Besides, the evaluation methods used to determine the efficacy of LTG were inconsistent, which may also have contributed to the varying recommended reference ranges. 2 researches [16, 20] assessed the efficacy of LTG based on the average monthly number of epileptic seizures over a period of 3 months, while 1 study [19] used the average monthly number of seizures over 28 days, the time frame used in other studies was unclear.

### Estimation of reference range

The reference range is defined as a range where clinical response is relatively unlikely to occur when the concentration is below the lower limit, and risk of toxicity obviously increases when the concentration is above the upper limit [4, 6]. If possible, lower limit of reference range should be based on studies that estimated the association between concentration and clinical response, utilizing statistical methods such as ROC analysis and randomized controlled trials of fixed concentration can be helpful in establishing this range [21]. Compared to the latter, ROC analysis is more feasible and have been performed in several studies of ASMs reference range [22, 23]. For the upper limit, a concentration-toxicity histogram is commonly utilized. This method involves identifying the concentration threshold at which the incidence rate of toxicity increases rapidly [24, 25]. Additionally, ROC analysis can be performed when there is a positive correlation between drug concentration and adverse drug reactions [26]. However, data on the adverse drug reactions are often lacking, so upper limit of many ADEs were based on the threshold that therapeutic effect no longer obviously increase [6]. Besides, the enrolled studies have inconsistent methods for calculating upper limits, such as concentration-effect curves, mean plus standard deviation, 95% confidence interval and percentile interval. Therefore, it is essential to determine the optimal method for calculating upper limits in LTG.

### Significance of establishing a reference range for children

Children require special consideration for ASMs treatment as clinical response cannot be extrapolated from adult evidence [6, 27, 28]. Predicting the necessary dosage for children is more challenging than for adults [4, 29, 30]. Reference ranges for LTG are primarily based on adult studies and may not be applicable to children [24, 25, 31]. Besag et al. [32] conducted a study involving 176 patients with epilepsy and observed a significant increase in adverse effects when the blood level of LTG exceeded 15 mg/L. Similarly, Fröscher et al. [33] enrolled 15 adult patients with epilepsy and found that the frequency of adverse effects increased slowly within the reference range of 5-13 mg/mL, but showed a sharp increase above 13-14 mg/mL. Another study by Hirsch et al. [24] included 811 patients with epilepsy and proposed a reference range of 1.5–10 mg/L for LTG. In a retrospective analysis conducted by Morris et al. [31] in 1998, plasma concentrations of 149 patients with epilepsy were examined. The study suggested that a higher reference range of 3–14 mg should be applied in patients who have not achieved maximum clinical benefit with lower concentrations. In 2004, another study [34] conducted by the same authors reviewed the clinical application of the reference range of 3–14 mg/L. The results indicated that the reference range was clinically accepted and provided therapeutic benefits, with few long-term adverse effects reported. Based on the threshold concentration for effectiveness and safety, the TDM guidelines of ILAE [4] and AGNP [6] recommended the reference range of 2.5 or 3 mg/L to 15 mg/L for LTG in adult epilepsy treatment.

Interestingly, unlike studies conducted on adults, the upper limit of reference ranges for children in our enrolled studies were estimated solely based on concentration and efficacy data. None of the studies utilized safety data in their estimations, possibly due to challenges in collecting adverse drug reaction data, the insufficient samples of high blood concentrations in children, or differences in opinions regarding the definition of reference range. Thus, it’s difficult to determine whether the threshold concentration of safety in children is different from that in adults. For instance, Wang et al. [20] redefined the upper limit of the LTG reference range by determining the concentration beyond which clinical benefits no longer increase. The study concluded that a significant proportion of epilepsy patients were being overtreated, and blindly increasing dosage to achieve higher trough levels did not result in additional improvement in clinical response. Moreover, it potentially exposed patients to unnecessary adverse events. Therefore, the study recommended a reference range based on efficacy to avoid overtreatment. Other studies estimated the reference range by considering the concentration levels in the majority of cases. For instance, Li and Huang [14] and Bao et al. [13] defined the 95% confidence interval of concentrations across all cases and effective cases as the reference range respectively.

In our opinion, it is crucial to avoid overtreatment. However, it should be noted that the upper limit of the reference range, as estimated based on efficacy data, is generally much lower. These lower limits are more appropriate for non-refractory epilepsy and may restrict treatment options for children who require a higher concentration. Additionally, it has been observed that higher concentrations are often tolerated and may provide benefits for patients with refractory epilepsy [24]. Thus, it’s also necessary to establish a safety-based upper limit of reference range for children, beyond which the adverse effects significantly increase.

Although previous studies reported a lack of a clear-cut relationship between clinical response and LTG concentration [35], however, more recent studies have further confirmed the existence of these relationships. One of our enrolled study [20] performed a logistic regression analysis and results showed that higher concentrations of LTG predicted a higher probability of clinical response, and the ROC analysis also confirmed the value of targeted concentrations beyond a specific level. Another study [19] also found that in the cases of combination use with VPA, the blood levels of effective cases were significantly higher than ineffective cases. Additionally, several studies have demonstrated the correlation between concentrations and tolerability [24], and it was also demonstrated that the incidence of toxicity increases significantly with concentrations above 13-15 mg/L [32, 33]. Therefore, we support the value of targeted concentrations within a specific range, and it is necessary to establish a reference range for children based on both efficacy and safety data.

### Strengths and limitations

This study has several strengths. Firstly, to our knowledge, this is the first systemic review of clinical studies on LTG reference range for children, providing credible evidence for the individualization of LTG in epilepsy treatment for children. Secondly, the study also conducted an analysis and comparison of calculation methods and data types, and discussed potential reasons for discrepancies between various studies. We also identified current research gaps, laying the foundation for further research in this area.

Limitations also exist in our study. Firstly, the number of included studies were relatively limited, and the quality of enrolled studies were generally not high. Secondly, all studies included in our analysis were conducted exclusively in China or Japan, indicating that the representativeness of the patient sample were insufficient [9], and the reference range identified in this study may be more relevant to children in these countries. However, further evaluation is necessary to determine the applicability of these findings to children in other countries. Thirdly, most studies used a flexible dose rather than a fixed dose, which may give rise to artificially negative correlations between concentrations and clinical effects [9, 36]. Additionally, it is important to note that the concentration types analyzed in the Chinese studies were trough concentration, while the only study from Japan were peak concentration. Therefore, medical workers should consider the type of concentration measured before referring to the reference range determined in our study.

## Conclusion

In summary, various studies have attempted to establish reference ranges for LTG in the treatment of child epilepsy. For LTG monotherapy, reference range were basically consistent, the lower limit of reference range for older children is similar to that recommended for adult by ILAE and AGNP guidelines, and younger children may need higher concentrations than older children and adults. However, there still remains controversial for LTG that concomitant with VPA or with other ASMs. Furthermore, unlike studies conducted on adults, all the reference ranges that have been established are based solely on concentration and/or efficacy data, with no studies having estimated the upper limit of the range based on safety data. Further high-quality research is required to determine the reference ranges for LTG monotherapy and concomitant with VPA, taking into account both efficacy and safety data.

### Supplementary Information

Below is the link to the electronic supplementary material.

Supplementary file1 Supplementary table 1 Retrieval strategy of PubMed. (PDF 117 KB)

## Data Availability

Data sharing not applicable to this article as no datasets were generated or analysed during the current study.
