# Supplementary material for: The reference range of lamotrigine in the treatment of epilepsy in children: a systematic review
Source: Eur J Clin Pharmacol. 2023 Oct 31;80(1):1–10. doi: 10.1007/s00228-023-03562-9 (PMC10781876; doi:10.1007/s00228-023-03562-9)
Supplement: Supplementary file 1 — Supplementary file1 Supplementary table 1 Retrieval strategy of PubMed. (PDF 117 KB) [file 228_2023_3562_MOESM1_ESM.pdf]

The concentration reference range of lamotrigine in the treatment of epilepsy in children: a systematic review

Jingjing Chen, Liang Huang, Linan Zeng, Zhimei Jiang, Meiping Xiong, Zhi-Jun Jia, Guo Cheng, Liyan Miao, Limei Zhao*, Lingli Zhang*

Journal: European Journal of Clinical Pharmacology

***Correspondence:**
Lingli Zhang
Email: zhanglingli@scu.edu.cn
Affiliation: Department of Pharmacy, West China Second University Hospital, Sichuan University, Chengdu, China

Limei Zhao
Email: lmzhao19@163.com
Affiliation: Department of Pharmacy, Shengjing Hospital of China Medical University, Shenyang, China.

Supplementary table 1 Retrieval strategy of PubMed

#1 "Adolescent"[MeSH Terms] OR "Child"[MeSH Terms] OR "Infant"[MeSH Terms] OR "Pediatrics"[MeSH Terms] OR "adolescent*"[Title/Abstract] OR "child*"[Title/Abstract] OR "infant*"[Title/Abstract] OR "pediatric*"[Title/Abstract] OR "paediatric*"[Title/Abstract] OR "teenager*"[Title/Abstract]
#2 "Lamotrigine"[Mesh] OR Lamictal [Title/Abstract] OR Lamotrigine [Title/Abstract]
#3 ("therapeutic"[Title/Abstract] OR "optimal"[Title/Abstract] OR "effective"[Title/Abstract] OR "target"[Title/Abstract] OR "orienting"[Title/Abstract] OR "reference"[Title/Abstract] OR "desirable"[Title/Abstract]) AND ("range*"[Title/Abstract] OR "window*"[Title/Abstract] OR "concentration*"[Title/Abstract] OR "level*"[Title/Abstract] OR "value*"[Title/Abstract])
#4 "Epilepsy"[Mesh] OR Epileps*[Title/Abstract] OR Seizure*[Title/Abstract]
#5 #1 AND #2 AND #3 AND #4
